# Characterizing the genetic basis of copper toxicity in *Drosophila* reveals a complex pattern of allelic, regulatory, and behavioral variation

**DOI:** 10.1093/genetics/iyaa020

**Published:** 2020-12-10

**Authors:** Elizabeth R Everman, Kristen M Cloud-Richardson, Stuart J Macdonald

**Affiliations:** 1 Department of Molecular Biosciences, University of Kansas, Lawrence, KS 66045, USA; 2 Center for Computational Biology, University of Kansas, Lawrence, KS 66047, USA

**Keywords:** heavy metal resistance, copper, life stage-specific, DSPR, QTL, MPP

## Abstract

A range of heavy metals are required for normal cell function and homeostasis. However, the anthropogenic release of metal compounds into soil and water sources presents a pervasive health threat. Copper is one of many heavy metals that negatively impacts diverse organisms at a global scale. Using a combination of quantitative trait locus (QTL) mapping and RNA sequencing in the *Drosophila* Synthetic Population Resource, we demonstrate that resistance to the toxic effects of ingested copper in *D. melanogaster* is genetically complex and influenced by allelic and expression variation at multiple loci. QTL mapping identified several QTL that account for a substantial fraction of heritability. Additionally, we find that copper resistance is impacted by variation in behavioral avoidance of copper and may be subject to life-stage specific regulation. Gene expression analysis further demonstrated that resistant and sensitive strains are characterized by unique expression patterns. Several of the candidate genes identified via QTL mapping and RNAseq have known copper-specific functions (*e.g.*, *Ccs*, *Sod3*, *CG11825*), and others are involved in the regulation of other heavy metals (*e.g.*, *Catsup*, *whd*). We validated several of these candidate genes with RNAi suggesting they contribute to variation in adult copper resistance. Our study illuminates the interconnected roles that allelic and expression variation, organism life stage, and behavior play in copper resistance, allowing a deeper understanding of the diverse mechanisms through which metal pollution can negatively impact organisms.

## Introduction

Anthropogenic release of heavy metals into soil and water sources presents a pervasive threat with long-lasting ecological, health, and economic impacts (Wu *et al.* 1975; Babin-Fenske and Anand 2011; Wuana and Okieimen 2011; Gall *et al.* 2015). Elevated heavy metals have been reported in dozens of organisms at all levels of the ecosystem (Neuberger *et al.* 1990; Georgieva *et al.* 2002; Sánchez-Chardi and Nadal 2007; Usmani 2011; Gall *et al.* 2015; Wright *et al.* 2015; Ecke *et al.* 2017; Plessl *et al.* 2017; Ilunga Kabeya *et al.* 2018), demonstrating that heavy metal pollution is wide reaching and can spread through food webs (Gall *et al.* 2015; Ilunga Kabeya *et al.* 2018). Although required for normal physiological function at low concentrations, copper is one of many common environmental heavy metal pollutants linked to mining (Ramirez *et al.* 2005; Wuana and Okieimen 2011; Wright *et al.* 2015), drinking water pipes (Harvey *et al.* 2016), and pesticide and fertilizer use (de Oliveira-Filho *et al.* 2004; Wuana and Okieimen 2011). In its essential role, copper helps bind oxygen, catalyzes enzymatic reactions, and promotes normal neurological development (Hart *et al.* 1928; Danks 1988; World Health Organization *et al.* 1996; Uriu-Adams and Keen 2005; Norgate *et al.* 2006; Navarro and Schneuwly 2017). However, excessive copper exposure ultimately leads to the overproduction of reactive oxygen species (ROS), which can cause cellular damage through oxidative stress (Uriu-Adams and Keen 2005; Tchounwou *et al.* 2008).

Evolutionarily conserved metal-responsive transcription factor 1 (MTF-1) and metallothionein (MT) proteins function as a first line of defense against toxic effects of excessive copper exposure in diverse organisms including humans, flies, fungi, and plants (Macnair 1993; Goldsbrough 2000; Bellion *et al.* 2007; Calap-Quintana *et al.* 2017). MTF-1 binds to metal responsive elements of MT genes, increasing MT abundance in copper accumulating cells and allowing excess heavy metal ions to be sequestered until they are removed from the system (Filshie *et al.* 1971; Stuart *et al.* 1985; Mokdad *et al.* 1987; Egli *et al.* 2003; Balamurugan *et al.* 2004; Southon *et al.* 2004). Metal chaperone and transporter proteins such as *Ccs* (Culotta *et al.* 1997), *Atox1* (Southon *et al.* 2004; Hatori and Lutsenko 2013), *ATP7* (Norgate *et al.* 2006), and *CTR1* family transporters (Petris *et al.* 2003; Guo *et al.* 2004; Balamurugan *et al.* 2007; Turski and Thiele 2007; Calap-Quintana *et al.* 2017; Navarro and Schneuwly 2017) also play a crucial role in the response to heavy metal toxicity (Egli *et al.* 2003; Petris *et al.* 2003; Yepiskoposyan *et al.* 2006; Janssens *et al.* 2009). For example, in *Drosophila melanogaster*, high copper exposure decreases translation of *Ctr1A* and *Ctr1B* via *MTF*-*1* to reduce influx of copper ions (Balamurugan *et al.* 2007; Turski and Thiele 2007; Calap-Quintana *et al.* 2017; Navarro and Schneuwly 2017), whereas high copper exposure in humans leads to degradation of the *hCTR1* protein (the human ortholog of *Ctr1A/B*) and a reduction in the intracellular concentration of copper (Petris *et al.* 2003; Guo *et al.* 2004).

Much of our understanding of the response to copper stress has come from studies that use genetic manipulation to define the roles of metal responsive genes (*e.g.*, Egli *et al.* 2003, 2006a; Bellion *et al.* 2007; Kirby *et al.* 2008; Bahadorani *et al.* 2010). However, quantitative trait locus (QTL) and GWA (genome-wide association) studies have demonstrated the genetic complexity of the response to heavy metal stress (Courbot *et al.* 2007; Willems *et al.* 2007; Zhou *et al.* 2017). For example, QTL mapping using *Caenorhabditis elegans* recombinant inbred advanced intercross lines showed that several regions of the genome are involved in the response to cadmium, copper, and silver exposure (Evans *et al.* 2018). GWA with the *Drosophila* Genetic Reference Panel (DGRP) revealed multiple candidate loci associated with the response to cadmium and lead stress (Zhou *et al.* 2017). QTL mapping of metal resistance in plants has further demonstrated the role that allelic variation plays in the response to heavy metal stress (Courbot *et al.* 2007; Willems *et al.* 2007; Turner *et al.* 2010; Arnold *et al.* 2016). For instance, an interspecific QTL study of two closely related species of *Arabidopsis* (metal-tolerant *A. halleri* × metal-sensitive *A. lyrata petraea*) identified multiple regions of the genome that contributed to zinc and cadmium resistance, and demonstrated that metal resistant alleles had become fixed in the metal tolerant species in the populations sampled (Courbot *et al.* 2007; Willems *et al.* 2007). Similarly, sequencing of *A. arenosa* populations locally adapted to serpentine soils revealed strong selection for introgressed alleles from the more tolerant *A. lyrata* (Arnold *et al.* 2016). These and other examples from *Mimulus gutattus* growing in copper mine tailings (Allen 1971; Macnair 1983; MacNair *et al.* 1993; Wright *et al.* 2015; Selby and Willis 2018) highlight the utility of using a quantitative genomics approach with powerful mapping panels to examine the influence of allelic variation on metal tolerance.

*Drosophila melanogaster* is an important model for understanding the mechanisms involved in the response to toxic heavy metal exposure due to the ease with which it can be genetically manipulated (*e.g.*, Egli *et al.* 2006a; reviewed in Navarro and Schneuwly 2017) and because of the extensive conservation of genes involved in the response to metal ions between flies and humans (Calap-Quintana *et al.* 2017). In addition, the existence of large *Drosophila* mapping panels makes this model system especially well-suited for examining the effect of naturally occurring alleles on the response to heavy metal stress. Finally, since *D. melanogaster* is a widespread species that is closely associated with human activities, and is commonly found in agricultural settings (Keller 2007; *e.g.*, Gleason *et al.* 2019) where heavy metal-containing pesticides, fungicides, and fertilizers may be in use, understanding the response to heavy metal exposure in flies has potential ecological and agricultural relevance.

In this study, we used more than 1500 strains from the *Drosophila* Synthetic Population Resource (DSPR) (King *et al.* 2012a, 2012b) to investigate the influence of allelic variation on the response to toxic copper exposure through QTL mapping, used RNA sequencing of copper-resistant and copper-sensitive strains to assess changes in gene expression following copper exposure, and followed up several plausible candidate genes with RNAi. Since a number of genes are known to respond to multiple heavy metals (Calap-Quintana *et al.* 2017), and pleiotropic QTL can underlie genetic variation for multiple metal resistance traits (Evans *et al.* 2018), our findings on the genetic architecture of copper resistance have the potential to provide broader insight into the allelic and expression variation influencing heavy metal stress.

## Materials and methods

### Mapping panel

We reared and phenotyped the >1500 recombinant inbred lines (RILs) that comprise the DSPR to measure variation in susceptibility to copper stress. The DSPR is a multiparental, advanced generation intercross mapping panel derived from 15 unique and fully sequenced founder strains, which represent a global sampling of genetic diversity in *D. melanogaster*. The DSPR consists of two mapping panels (A and B), which are composed of two subpanels (A1 and A2, and B1 and B2). The subpanels were started from the same set of founders, but were maintained independently [see King *et al.* (2012b) for additional details on the mapping panel].

### Rearing and assay conditions

Strains from the DSPR were maintained, reared, and tested in the same incubator under a 12:12 h light:dark photoperiod at 25°C and 50% humidity. To obtain female flies for the adult copper resistance assay, RNA sequencing, and RNAi validation, adults were transferred to cornmeal–molasses–yeast food, allowed to oviposit for two days, then discarded. Experimental female, presumably mated, flies from the following generation were sorted over CO_2_ and placed into vials with new cornmeal–molasses–yeast media for 24 h before they were transferred to copper-supplemented food. All adult assays were performed on 3- to 5-day-old individuals.

### Adult female copper resistance

The adult female response to copper stress was measured as percent survival on media containing 50 mM CuSO_4_ following a 48-h exposure period. As essentially no flies die on control food (Highfill *et al.* 2016) or under starvation conditions (Everman *et al.* 2019) throughout the span of our assay, we did not assess adult female survival on control food in this study. Experimental females were transferred without CO_2_ anesthesia to vials containing 1.8 g potato-based Instant *Drosophila* Medium (Carolina Biological Supply Company 173200) hydrated with 8 mL 50 mM CuSO_4_ [Copper(II) sulfate, Sigma–Aldrich C1297]. Instant *Drosophila* Medium is estimated to contain approximately 0.02 mM Cu prior to hydration (Maroni and Watson 1985). Copper resistance was measured in a total of 11 batches across the A (N strains = 767) and B (N strains = 789) DSPR mapping panels. Each strain was measured in a single batch with three vial replicates each containing between 7 and 20 individuals (average number of flies per vial replicate = 19.4). The effect of copper on survival was reported as mean percent survival per strain across the three replicate vials. Retaining vials with fewer than 15 flies did not impact our QTL mapping results in a meaningful way (see below). Hereafter, the adult survival response to 48 h, 50 mM CuSO_4_ is referred to as adult copper resistance.

### Adult female feeding response to copper-supplemented media

A subset of strains evenly sampled throughout the B2 subpanel adult copper resistance distribution (0% ± 0 S.E.–98.4% ± 1.59 S.E.) were used to measure the effect of copper exposure on food intake. We measured food intake in three blocks with at least two vial replicates of 20 females per strain (*N* = 95) per treatment (control *vs* copper) with vial replicates distributed across blocks, following a protocol modified from (Shell *et al.* 2018). Briefly, we added 1% erioglaucine disodium salt (Sigma–Aldrich 861146), a blue dye, to water and to 50 mM CuSO_4_ no more than 24 h prior to the assay to avoid dye decomposition. We hydrated 0.9 g Instant *Drosophila* Medium with 4 mL liquid, and flies were allowed to consume dyed food for 24 h before they were frozen for up to 5 h. No flies died during the 24-h period. Subsequently, flies were homogenized with three to four glass beads in 600 µL distilled water for 45 s using a Mini-Beadbeater-96 (BioSpec Products). Homogenate was centrifuged for 5 min at 14,000 rpm, and 200 µL supernatant was transferred to a 96-well plate. Fly homogenate was frozen for up to 48 h before absorbance at 630 nm was measured with a BioTek Multimode Microplate reader (Synergy 2 v.1.03). Two water blanks and 14 standards ranging from 6.25 × 10^−5^% to 0.006% dye in water were prepared fresh for each block and were included in each plate to determine the dye concentration of fly homogenate, and to assess consistency among blocks. Absorbance readings for standards were highly correlated across plates and blocks (Supplementary Table S1). To calculate the estimated amount of dye consumed, we used a linear model to find the slope and intercept of the standard curve (Concentration of Standard ∼ Absorbance × Block). Estimated percent dye in each fly homogenate sample corrected for block variation observed among standards was determined with the equation:

$$\%\;\mathrm{Dye}\;\mathrm{in}\;\mathrm{sample}=\left(0.002443\;\times \;\mathrm{absorbance}\right)-0.0001465.$$

Variation in feeding behavior among DSPR strains on copper and control food was assessed with a two-way ANOVA with an interaction (% Dye Consumed ∼ DSPR Strain × Treatment), and effect size was calculated using Cohen’s F (R package sjstats) (Cohen 1988; Lüdecke 2018). The correlations between feeding behavior (average percent dye consumed per RIL) on copper and control food and adult copper resistance were assessed with linear models (% Dye Consumed ∼ Adult Copper Resistance). Feeding plasticity was calculated as the percent dye consumed on control food minus the percent dye consumed on copper-supplemented food, and the correlation between feeding plasticity and adult copper resistance was tested with a linear model (Feeding Plasticity ∼ Adult Copper Resistance).

### Developmental response to copper

Developmental viability was estimated in the B panel from 100 strains that were evenly sampled from throughout the distribution of B1 and B2 subpanel adult copper resistance (0% ± 0 S.E.–98.4% ± 1.59 S.E.). Approximately 100 females per strain were allowed to oviposit on cornmeal–molasses–yeast media for 17–20 h in 6 oz polypropylene *Drosophila* bottles (Genesee Scientific: 32-130) with yeast paste to encourage egg laying. Following oviposition, remaining yeast paste was removed, and embryos were gently dislodged from the media surface by rinsing with 1× PBS and swirling with a small, bristled paintbrush. Subsequently, for each strain, we arrayed multiple 10 µL aliquots of embryos suspended in 1× PBS onto a petri dish containing 2% agar dyed blue with Erioglaucine Disodium Salt (Sigma–Aldrich 861146; 8 mg/mL). For each dish, we aliquoted eggs into 14 cells (Supplementary Figure S1), photographed the dish (Nikon D3200, 105 mm 1:2.8 DG Sigma Macro lens), and the number of embryos within each cell was recorded with ImageJ (v. 1.51s). Embryos from each cell (30–306 embryos, average = 125 embryos) were then transferred with a rubber, bristleless paintbrush to vials containing control or 2 mm CuSO_4_ hydrated Instant *Drosophila* Medium (1.8 g media plus 8 mL liquid). The rubber paintbrush was examined after each egg transfer to ensure all eggs had been transferred to the vial. The developmental response to copper was assessed with 4–7 replicates per treatment for each strain (mean replicates per strain = 6.8). We used a lower copper concentration in this assay because previous studies have shown that the larval life stage is much more susceptible to copper toxicity compared to adults (Bahadorani and Hilliker 2009).

Copper stress has the potential to increase development time and reduce the number of individuals that complete development from egg to adult. To estimate the effect of copper exposure on development time, for each experimental vial, we recorded the number of days between set up and the first emergence of adults. We acknowledge this measure limits our assessment of the distribution of adult emergence times per vial, but we found that removing adult flies throughout the assay disrupted developing pupae, potentially impacting subsequent emergence counts. To assess the effect of copper on developmental viability, we calculated the proportion of embryos in each vial that eclosed as adults in the seven days following the day of first emergence. Developmental viability was square root transformed to improve deviation from normality within treatment (Shapiro–Wilks test on transformed data; *W* = 0.99, *P* = 0.04). From here forward, square-root transformed developmental viability is simply referred to as developmental viability, and all subsequent analyses were performed on square-root transformed data. Vials were monitored daily for 30 days after set up. Of the 1356 vials included in this assay, 100 copper treatment vials yielded zero flies. These vials were given a development time of 30 days.

We used a two-way ANOVA with an interaction to measure the effect of strain and treatment on each developmental trait. The DSPR strains we used in this study varied in development time (*F*_99,579_ = 11.8, *P* < 0.00001; Supplementary Table S2A) and developmental viability on control food (*F*_(99,579)_ = 31.6, *P* < 0.00001; Supplementary Table S2B). Furthermore, regression analysis demonstrated that development time and developmental viability in control and copper conditions were correlated (development time: *F*_(1,98)_ = 61.0, *P* < 0.0001, *R*^2^ = 38%; Supplementary Table S2C and Figure S2A; developmental viability: *F*_(1,98)_ = 54.1, *P* < 0.0001, *R*^2^ = 36%; Supplementary Table S2D and Figure S2B). Therefore, we employed linear models to regress out variation in control development time and control developmental viability to more directly assess the effect of copper stress on these metrics of development. Residual development time and residual developmental viability are referred to from hereafter as treatment-specific development time and treatment-specific developmental viability, respectively.

### Heritability

We estimated the genetic and phenotypic variances of adult copper resistance, control and copper feeding responses, treatment-specific development time, and treatment-specific developmental viability using linear mixed models (lme and varcomp functions in R; R package: APE, Paradis *et al.* 2004; R package: nlme, Pinheiro *et al.* 2017). For all responses, panel-specific broad-sense heritabilities were calculated as the proportion of the total strain-specific variation explained by the estimated genetic variance component (Lynch and Walsh 1998).

### QTL mapping of life stage-specific response to copper stress

We used the DSPRqtl package in R (github.com/egking/DSPRqtl; FlyRILs.org) to identify QTL associated with variation in all measured traits. QTL mapping was performed for each mapping panel (A and B) and phenotype separately. At each position in the genome, for each strain, we can estimate the additive probability that the segment of the genome was inherited from each of the eight DSPR founders. QTL were identified by regressing the strain mean phenotype on these probabilities, and significance thresholds were assigned following 1000 permutations of the data (King *et al.* 2012a, 2012b). For adult copper resistance, peak positions for each QTL were estimated with 2-LOD support intervals (King *et al.* 2012a). Because fewer strains were used to measure the feeding and treatment-specific development traits, to better approximate a 95% confidence interval on QTL position, we used a 3-LOD drop (King *et al.* 2012a). Using a 3-LOD drop inevitably has the effect of broadening the QTL interval, which may increase the likelihood of detecting overlap among QTL mapped for different traits, and lead to a spurious inference of pleiotropy. However, we found no difference in the number of QTL that overlapped among traits when employing 2- or 3-LOD drops, and therefore only present results based on 3-LOD drops below. We performed gene ontology (GO) analysis without normalizing for gene length for genes included in peak intervals for each trait and mapping panel separately (FlyMine.org; Lyne *et al.* 2007), but saw no GO enrichment likely because QTL intervals include many non-causative genes that potentially obscure any signal of enrichment.

Adult copper resistance varied between the A1 and A2 subpanels but did not vary between the B subpanels (A panel: *F*_1,2289_ = 12.64; *P* < 0.001; B panel: *F*_1,2495_ = 0.03; *P* = 0.86; Supplementary Figure S3 and Table S2, E–F). Therefore, subpanel was included as a model covariate only in the QTL analysis of panel A. Phenotyping batch also significantly influenced variation in adult copper resistance in both the A and B panels (Supplementary Table S2, E–F). Because batch and subpanel were confounded, we could not test the effects of both covariates on adult copper resistance at the same time. However, including batch as a covariate in the QTL mapping model did not drastically alter the estimation of LOD scores for either panel (A panel correlation between LOD scores = 99%; B panel correlation = 98%; Supplementary Figure S4A) or the presence of any QTL, so we only present data from the models lacking batch as a covariate. Because the development assay was conducted on 100 strains across 15 batches, DSPR strain was highly confounded with batch. Therefore, we did not include batch or subpanel as a covariate in the QTL mapping models for treatment-specific development time or treatment-specific developmental viability. As each strain assessed in the feeding response assay was measured in each of three blocks, block could not be included in the model for either average feeding response on control or copper food.

To determine whether including vials containing relatively few flies influenced QTL mapping results due to mis-estimated phenotype means, we additionally mapped variation in adult copper resistance using only data from vials containing at least 15 flies (removing 316 or 7% of the vials). LOD scores for the full data set were highly correlated with those for the reduced dataset for each panel (A panel correlation = 99%; B panel correlation = 99%; Supplementary Figure S4B) and the same QTL were identified in each analysis, so we only present data from analyses using all vials.

### QTL-centered association tests

Since all DSPR founders are sequenced, we can use the founder haplotype structure of each RIL to infer the allele that each RIL possesses at each variant segregating among the founders. We can then fit a single marker model at each variant beneath a mapped QTL to associate phenotype and genotype, and examine whether individual variant sites can explain QTL peaks (see Kislukhin *et al.* 2013; King *et al.* 2014; Marriage *et al.* 2014 for more detail). In principle, executing such analyses can help resolve, or fine-map QTL identified in multiparental mapping populations (see for instance Figure 5 from Gatti *et al.* 2014).

### Differential gene expression in high and low adult copper resistance strains

We examined gene expression variation in a subset of 10 strains (six with high adult resistance: 76–98% survival, and four with low adult resistance: 0.0–18% survival) from the B panel to explore how adult copper resistance class and gene expression interact when individuals are exposed to 50 mM CuSO_4_. Twenty experimental females from each DSPR strain were transferred to Instant *Drosophila* Medium hydrated with either water as a control or 50 mM CuSO_4_ (the same concentration used for the adult resistance assay) for 9 h. No individuals died during the 9-h exposure period. Following exposure, 10 females from each strain and treatment were flash frozen in liquid nitrogen, placed in TRIzol Reagent (Invitrogen, 15596018), and immediately stored at −80°C. RNA was extracted from each of the 20 samples with the Direct-zol RNA Miniprep kit (Zymo Research, R2050), eluted in 100-µL water, and stored at −80°C. We prepared libraries with the TruSeq Stranded mRNA kit (Illumina, 20020595), and paired-end 37-bp mRNA libraries were each sequenced to ∼20 million reads on an Illumina NextSeq 550 at the University of Kansas Genome Sequencing Core.

Sequence quality assessment and trimming were performed using fastp (Chen *et al.* 2018). We used kallisto to perform pseudoalignment-based mapping of reads (Ensembl transcriptome release 90) (Bray *et al.* 2016), and performed differential expression analysis with sleuth (v.0.30.0) using likelihood ratio tests (Pimentel *et al.* 2017). Gene expression is likely to vary between the different DSPR strains; however, we were primarily interested in understanding whether there are consistent differences in gene expression between high and low resistance classes of strains. Given this interest, we treated each strain as a biological replicate of the high and low resistance classes and did not include DSPR strain in differential expression models. After determining that the interaction between resistance class and treatment did not influence expression of any gene at a 5% FDR (False Discovery Rate), we tested the additive effects of resistance class and treatment on gene expression, referred to from here as the full model (full model: ∼TRT + RES *vs* reduced model: ∼1). We also examined the influence of each term independently in two additional models. The effect of treatment alone was assessed by accounting for resistance class (treatment model: ∼TRT + RES *vs* reduced model: ∼RES), and the effect of resistance class alone was assessed by accounting for treatment (resistance model: ∼TRT + RES *vs* reduced model: ∼TRT). From here on, these term-specific models are referred to as the treatment model and the resistance model, respectively. Significantly differentially expressed (DE) genes for each model were identified with a 5% FDR threshold.

We generated three lists of significantly DE genes: full model DE genes, treatment model DE genes, and resistance model DE genes. Sleuth applies a filter against genes with low expression (Pimentel *et al.* 2017). We applied an additional filter following sleuth analysis to remove genes from DE gene lists with average expression of less than 1 TPM (Transcripts Per Million). Additionally, we eliminated genes with expression variance ≥1 TPM in any of the following four categories: sensitive strains, control treatment; sensitive strains, copper treatment; resistant strains, control treatment; resistant strains, copper treatment (Supplementary Figure S5). We used principal components analysis (PCA) to examine the effect of treatment and resistance class using quantile normalized TPM data for DE genes. DE gene lists were examined for co-regulated clusters of genes using Clust (Abu-Jamous and Kelly 2018). GO analysis was performed for each cluster and for each of the DE gene lists in their entirety (FlyMine.org; Lyne *et al.* 2007).

### RNAi knockdown of candidate genes associated with adult copper resistance

Several candidate genes were implicated by QTL mapping and/or RNAseq analysis of adult females. TRiP UAS-RNAi strains (Perkins *et al.* 2015) for candidate genes, as well as a control UAS-LUC.VALIUM10 strain, were obtained from the Bloomington Drosophila Stock Center (BDSC) (Supplementary Table S3). Crosses involved 10 TRiP males and 10 virgin females from Gal4-expressing driver strains. Each TRiP strain was crossed to both a ubiquitous Gal4 driver strain (BDSC 25374), with Gal4 under the control of the Act5C promoter, and a strain expressing Gal4 in the adult anterior midgut (1099 from Nicholas Buchon, flygut.epfl.ch; Buchon *et al.* 2013). Three candidate gene TRiP strains (*swm*, *Catsup*, and *CG11825*) produced too few flies to test when crossed to the ubiquitous Gal4-expressing driver and were thus excluded from our analysis. We tested two TRiP UAS RNAi strains for the genes *CG5235*, *MtnC*, and *ZnT41F* to assess the consistency in the effect of gene knockdown on copper survival (Supplementary Table S3).

An average of 19.3 Gal4-UAS RNAi females (min 7) were transferred to Instant Drosophila Medium hydrated with 50 mM CuSO_4_ using an average of 16.8 (min 10) replicate vials per genotype (a total of 203–365 individuals per genotype) across four batches. We counted flies daily until all were dead, and the response to copper stress in these RNAi knockdown genotypes was quantified as average lifespan. We chose to measure lifespan on copper instead of percent survival at 48 h (as in our DSPR screen) because we had no *a priori* expectation that survival would be variable at 48 h among the RNAi genotypes, and knockdown in genes hypothesized to influence the response to copper toxicity could drastically reduce or extend survival. To establish that GAL4-UAS-RNAi genotypes were not inherently unhealthy, we additionally placed 20 such females from each cross on Instant *Drosophila* Medium hydrated with water to assess overall viability. No individuals died on copper-free media through the duration of the RNAi assay. We compared copper resistance for each RNAi knockdown to its respective control using per vial average lifespan controlling for batch with a two-way ANOVA (Average Lifespan ∼ Strain × Batch) with planned comparisons. These analyses were performed separately for each GAL4 driver.

### Data availability

All raw data and images generated from this study, including adult and developmental copper resistance traits, feeding data, raw QTL mapping data, normalized TPM expression data, and RNAi data are available at FigShare. Supplementary File S1 contains descriptions for all accompanying data files. DSPR genotype information is publicly available at www. FlyRILs.org. RNAseq reads are available from NCBI SRA PRJNA633166. Unless otherwise stated, all analyses were performed in R (v. 3.6.2) (R Core Team 2017).

Supplemental material is available at figshare DOI: https://doi.org/10.25386/genetics.13283981.

## Results

### Abundant variation in adult female copper resistance

**Figure 1:**
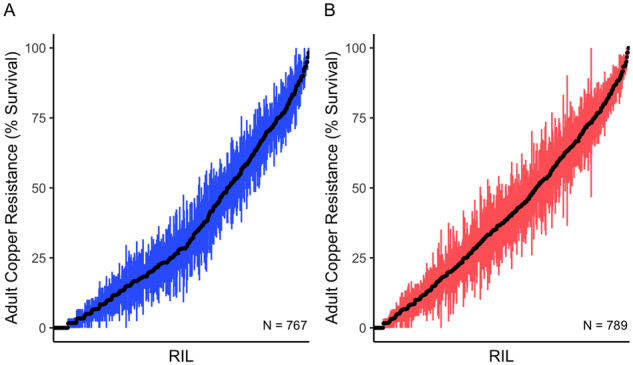
Adult copper resistance is highly variable in the DSPR. Variation in mean female adult copper resistance (±S.E.) per DSPR strain in (A) and (B) panels following 48-h exposure to 50 mM CuSO_4_. Recombinant inbred lines (RILs) are ordered by phenotype along the *x* axis.

We measured adult copper resistance in females from over 1500 DSPR RILs by exposing 60 flies (3 vials of 20 flies) from each strain to 50 mM CuSO_4_ for 48 h. Phenotypic variation and heritability were high for female copper resistance in both the A and B panels of the DSPR (A panel: *H*^2^ = 83.0%; B panel: *H*^2^ = 78.8%; Figure 1).

### Adult female feeding response to copper-supplemented media

**Figure 2:**
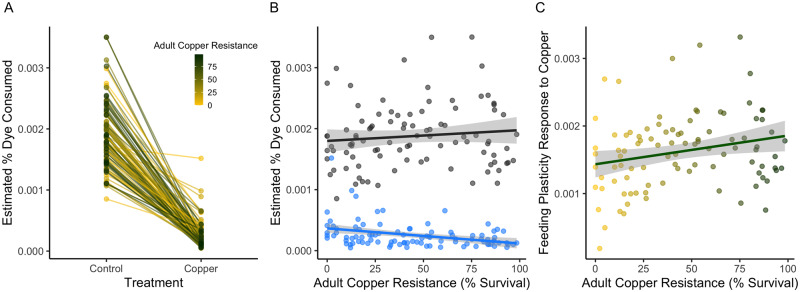
Feeding aversion to copper varies in the DSPR. Feeding behavior in 95 DSPR strains changed in response to 24-h exposure to 50 mM CuSO_4_. (A) Mean percent dye consumed varied among DSPR strains (*P* < 0.00001) and was much lower under copper conditions relative to control (water) conditions (Treatment: *P* < 0.00001). The interaction between strain and treatment (*P* < 0.00001) suggests that the feeding response to copper varies among the strains. (B) Feeding behavior under control conditions was not correlated with adult copper resistance (*P* = 0.32); feeding behavior on copper was correlated with adult copper resistance (*P* = 0.0006). Feeding response to copper is shown in blue; the control response is shown in black. (C) Feeding plasticity (calculated as the difference in dye consumption in control and copper food) was correlated with adult copper resistance (*P* = 0.02), suggesting that resistant strains displayed greater behavioral plasticity in response to copper compared to sensitive strains. Point color indicates adult copper resistance, as in (A). Shading around the regression in (B) and (C) indicates the 95% CI of the regression.

Since the toxicity of copper in our assay likely stems from ingestion, we were interested in flies’ feeding response to copper-supplemented media. Using a sample of 95 strains from the B2 subpanel that spanned the distribution of adult copper resistance (from 0% to 98.4% survival), we tested the effect of 50 mM CuSO_4_ on feeding behavior. We estimated feeding by measuring the amount of dye consumed by flies exposed to food hydrated with water or a copper solution within a 24-h period. Both DSPR strain and treatment significantly influenced feeding (DSPR Strain: *F*_94,384_ = 3.08, *P* < 0.00001; Treatment: *F*_1,384_ = 2306, *P* < 0.00001, Supplementary Table S2G; Figure 2A), although treatment had a much larger effect on the feeding response than strain (Cohen’s F: Treatment = 2.57, DSPR strain = 0.91). We also observed an interaction between strain and treatment (DSPR Strain × Treatment: *F*_94,384_ = 2.77, *P* < 0.00001, Cohen’s *F* = 0.86, Supplementary Table S2G), indicating that the reduction in feeding due to copper is not uniform across strains (Figure 2A). Both feeding responses had high heritability (control feeding response: *H*^2^ = 87.6%; copper feeding response: *H*^2^ = 87.6%).

Overall, feeding behavior under copper conditions was negatively correlated with adult copper resistance (*R* = −34.6%, *F*_1,93_ = 12.7, *P* = 0.0006; Supplementary Table S2H, Figure 2B), while feeding behavior under control conditions was not correlated with adult copper resistance (*R* = 10.2%, *F*_1,93_ = 0.98, *P* = 0.32; Supplementary Table S2I, Figure 2B). Feeding plasticity (calculated as the difference in percent dye consumed on control and copper media) was weakly positively correlated with adult copper resistance (*F*_1,93_ = 5.33, *P* = 0.02, *R*^2^ = 0.5%; Supplementary Table S2J), suggesting that copper resistant strains display greater behavioral plasticity in response to copper-supplemented food compared to sensitive strains (Figure 2C). Together, these results suggest that our adult copper resistance phenotype is partially influenced by a copper-induced behavior, where more sensitive strains tend to eat more copper food than more resistant strains in a 24-h period. Equally, the limited strength of the relationship likely implies our resistance phenotype is primarily impacted by the physiological and metabolic response to copper and is not solely influenced by behavioral avoidance.

### Developmental response to copper

**Figure 3:**
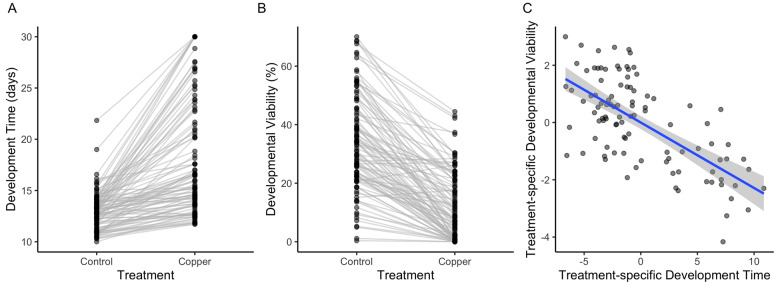
Copper exposure influences development time and viability in the DSPR. Development time (A) and developmental viability (B) were reduced in most strains by exposure to 2 mM CuSO_4_. (C) Copper treatment-specific developmental viability and development time (corrected for strain-specific variation in these responses on control food) were correlated (*P* < 0.00001, *R*^2^ = 44%), indicating that strains with longer development time on copper-supplemented media also had lower viability. Points indicate the strain mean under each treatment condition. Gray shading indicates the 95% CI of the regression.

In organisms with complex life cycles, the genetic control of physiological traits can be decoupled between life stages (Freda *et al.* 2017; Collet and Fellous 2019). To assess whether the strains with high resistance to copper as adults were also more resistant in other life stages, we sampled 100 strains from the B1 and B2 subpanels that span the range of adult copper resistance (from 0% to 98.4% survival). Embryos from these strains were placed on control media or media containing 2 mM CuSO_4_, and the day of first adult emergence (development time) and the proportion of embryos that emerged as adults (developmental viability) were recorded. Both development time and developmental viability were variable among strains on copper and control media (development time: *F*_99,1157_ = 24.21, *P* < 0.00001; developmental viability: *F*_99,1157_ = 49.17.21, *P* < 0.00001; Supplementary Table S2, K–L, Figure 3). Exposure to copper delayed emergence by nearly 4 days on average (*F*_1,1157_ = 1293, *P* < 0.00001; Supplementary Table S2K, Figure 3A) and significantly reduced developmental viability (*F*_1,1157_ = 3905, *P* < 0.00001; Supplementary Table S2L, Figure 3B). There was an interaction between strain and treatment for both measures of the developmental response to copper, indicating that although development time and developmental viability were negatively affected by copper exposure for the majority of strains, the magnitude of the effect of treatment varied among strains (development time: *F*_1,1157_ = 11.75, *P* < 0.00001; developmental viability: *F*_1,1157_ = 13.13, *P* < 0.00001; Supplementary Table S2, K–L, Figure 3, A and B).

Because we were primarily interested in the effects of copper on development time and developmental viability, we regressed out variation under control conditions from both developmental phenotypes (see *Materials and Methods*). Treatment-specific development time and treatment-specific developmental viability were correlated (*F*_1,98_ = 76.4, *P* < 0.00001, *R*^2^ = 44%; Supplementary Table S2M, Figure 3C), demonstrating that strains with longer development time on copper also had lower viability. Heritability was similar between treatment-specific development time (*H*^2^ = 87.7%) and treatment-specific developmental viability (*H*^2^ = 87.7%).

**Figure 4:**
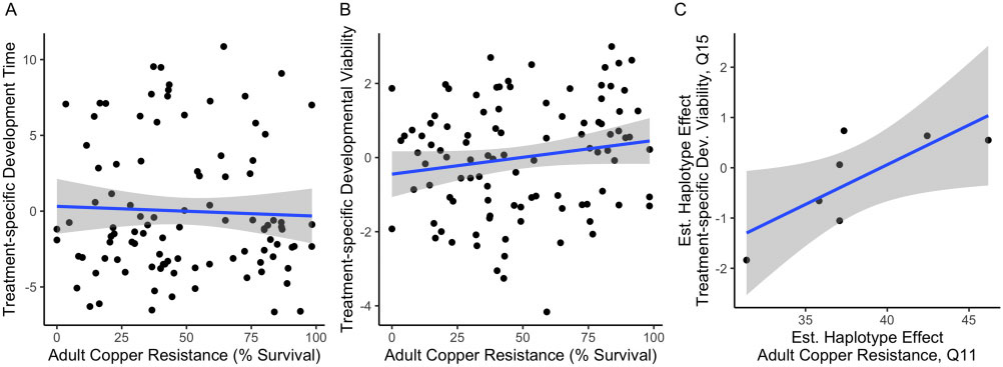
Copper resistance is not significantly correlated across life stages. (A) Copper treatment-specific development time was not correlated with adult copper resistance (*P* = 0.69). (B) Copper treatment-specific developmental viability and adult copper resistance were not correlated (*P* = 0.10). In both plots, points indicate strain means. Higher, positive values for treatment-specific development time and developmental viability indicate longer or higher development time or viability on copper, respectively. Gray shading indicates the 95% CI of the regression between residual developmental response (corrected for variation in the response on control food) and adult female survival after 48 h on 50 mM CuSO_4_. (C) Founder haplotype effects for copper treatment-specific developmental viability and adult copper resistance estimated at a shared QTL position on chromosome 3R were significantly positively correlated (*P* = 0.04, *R*^2^ = 59%). Gray shading indicates the 95% CI of the regression between estimated founder haplotype effects at Q11 for adult copper resistance and at the equivalent genomic position for treatment-specific developmental viability, which resides within Q15.

Neither treatment-specific development time nor treatment-specific developmental viability were correlated with adult copper resistance at an alpha level of 0.05 (treatment-specific development time: *F*_1,98_ = 0.16, *P* = 0.69, *R*^2^ = 0.02; Supplementary Table S2N, Figure 4A; treatment-specific developmental viability: *F*_1,98_ = 2.71, *P* = 0.10, *R*^2^ = 2.7%, Supplementary Table S2O, Figure 4B). The lack of a significant correlation between either measure of the developmental response to copper and adult copper resistance could imply that copper resistance is influenced by life stage-specific mechanisms. However, because several other aspects of our adult and development assays differ (*e.g.*, copper concentration and exposure time), we cannot rule out the possibility that the pattern we observe is also influenced by technical variation.

### QTL mapping of life stage-specific response to copper stress

**Figure 5:**
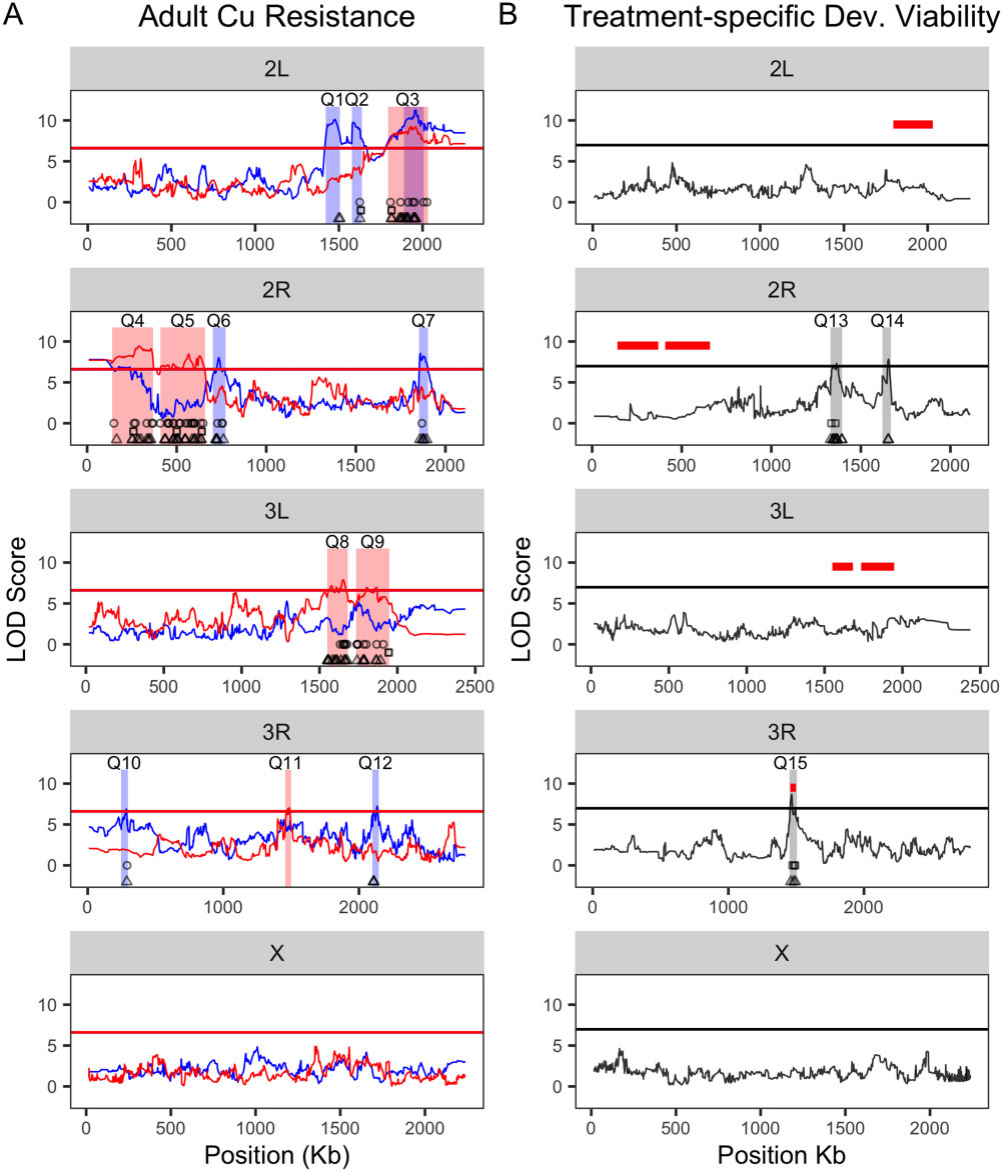
QTL associated with adult copper resistance and treatment-specific developmental viability. QTL associated with variation in adult copper resistance (A) and copper treatment-specific developmental viability (B). (A) We detected several QTL in the A (blue) and B (red) DSPR panels. Most QTL were panel specific with one QTL (Q3) overlapping between panels. Red and blue bars represent the 2-LOD drop intervals for each QTL. (B) QTL mapped for copper treatment-specific developmental viability. One QTL (Q15) for developmental viability overlapped with Q11, associated with adult copper resistance in the (B) panel. Red horizontal lines represent the 2-LOD drop intervals for the six QTL associated with the (B) panel adult survival response to copper, and gray bars represent the 3-LOD drop for the three QTL associated with treatment-specific developmental viability (Table 1). The horizontal lines in each plot represent permutation-derived 5% critical thresholds (the thresholds for each panel in (A). are nearly identical, leading to the lines overlapping.) Round points indicate DE genes influenced by resistance class, triangle points indicate DE genes influenced by treatment, and square points indicated DE genes that are shared between the treatment and resistance class models and that fall within the QTL boundaries. DE genes across the rest of the genome are not shown.

A principal goal of our study was to genetically dissect the response to copper stress. Using the DSPR, we identified a total of 12 QTL between the A and B panels that were associated with variation in adult copper resistance (Figure 5A, Table 1, and Supplementary Figure S6). Assuming that each QTL contributes to phenotypic variation in an additive manner, they collectively explain a substantial amount of variation in adult copper resistance (A panel: 36.19%; B panel: 27.93%; Table 1). The genetic architecture of adult copper resistance was largely panel-specific, with only one QTL (Q3) overlapping between the mapping panels (Table 1, Figure 5A). Panel-specific genetic architecture of trait variation is consistent with several other studies that have mapped traits in both panels of the DSPR (Marriage *et al.* 2014; Najarro *et al.* 2017; Everman *et al.* 2019). This pattern is likely the result of using a different set of founders to establish each mapping panel (King and Long 2017) but may also reflect a lack of power (King *et al.* 2012a) or epistatic effects that influence our ability to detect all QTL underlying adult copper resistance in each panel. Several pairs of QTL are in close proximity (*i.e.*, Q1/Q2, Q4/Q5, and Q8/Q9—Figure 5A), with 0.7–0.53 MB separating the QTL intervals. However, the founder haplotype effects at these QTL pairs appear distinct, and there is no evidence for radical shifts in haplotype frequency between the QTL (Supplementary Figures S7–S9), so we consider them independent.

**Table 1:** Summary of QTL identified for response to copper stress by panel and life stage

Adult copper resistance QTL: A panel						
QTL	Peak LOD	Chr	Physical interval (Mb)^a^	Genetic interval (cM)	Variance explained	No. genes^b^
Q1	10.13	2L	14.22–15.07	49.52–60.62	5.91	73
Q2	9.76	2L	15.77–16.38	51.35–51.88	5.70	68
Q3A	11.3	2L	18.87–20.09	53.39–53.85	6.56	154
Q6	8.01	2R	7.03–7.72	63.56–64.89	4.70	108
Q7	8.54	2R	18.54–19.03	100.24–102.17	5.01	92
Q10	6.88	3R	2.48–2.95	47.56–47.74	4.05	40
Q12	7.24	3R	21.01–21.46	86.55–88.14	4.26	78

Adult copper resistance QTL: B panel						

QTL	Peak LOD	Chr	Physical interval (Mb)^a^	Genetic interval (cM)	Variance explained	No. genes^b^

Q3B	9.21	2L	17.95–20.31	52.92–53.90	5.24	259
Q4	9.44	2R	1.41–3.69	54.99–57.07	5.36	247
Q5	8.46	2R	4.08–6.58	57.77–62.69	4.82	353
Q8	7.88	3L	15.50–16.82	42.90–44.06	4.49	240
Q9	6.97	3L	17.35–19.47	44.48–45.78	3.99	224
Q11	7.05	3R	14.57–14.98	63.92–65.00	4.03	41

Treatment-specific developmental viability QTL: B panel						

QTL	Peak LOD	Chr	Physical interval (Mb)^a^	Genetic interval (cM)	Variance explained^c^	No. genes^b^

Q13	7.11	2R	13.30–13.95	82.37–84.50	27.9	125
Q14	7.74	2R	16.19–16.66	90.86–92.45	30.0	60
Q15	8.65	3R	14.52–15.06	63.79–65.21	32.8	62

a Physical intervals are based on FlyBase release 5 of the *D. melanogaster* reference genome.

b Protein-coding genes only. All genes including ncRNA and pseudogenes are included in Supplementary Table S4.

c Estimates of QTL effects based on 100 DSPR strains are typically overestimated due to Beavis effects (Beavis *et al.* 1991; King and Long 2017), so estimates of the variance explained by the QTL mapped for developmental viability should be interpreted with care.

We did not find any QTL that contributed to food consumption, either on control or copper-supplemented food, or treatment-specific development time, even when using a relaxed (α = 0.2) significance threshold. However, we did find three QTL that contributed to variation in treatment-specific developmental viability (Figure 5B). Given we only phenotyped 100 DSPR strains, power deficits certainly contribute to the low numbers of QTL identified for these traits (power to detect a QTL that explains 10% of phenotypic variation with 100 DSPR strains is <20%) (King *et al.* 2012a).

One treatment-specific developmental viability QTL (Q15) overlapped with a QTL (Q11) associated with adult copper resistance in the B panel (Figure 5B). The 2-LOD drop interval of Q11 fell entirely within the 3-LOD drop interval of Q15. To determine whether the QTL may represent the same locus, we compared the founder effects for both phenotypes at the peak position of the Q11 adult QTL (considering that the Q15 developmental QTL peak may not be well estimated given the sample size employed). We found that the estimated founder effects were significantly positively correlated (*F*_1,5_ = 7.11, *P* = 0.04, *R*^2^ = 59%; Supplementary Table S2P, Figure 4C), suggesting this position segregates for alleles that impact the response to copper stress in adults and developing individuals in a similar way. This result implies that the adult and developmental response to copper stress are not fully independent, as was suggested by the very weak phenotypic correlation between these traits (Figure 4, A and B). The level of overlap we observed between the adult and developmental viability responses may also be impacted by low sample size; however, founder haplotype frequencies in the full set of strains and the subset of 100 strains used to measure the developmental viability response are very similar across the genome (Supplementary Figure S10), suggesting that the subset of lines captures the same allelic diversity present in the full set.

### Genes implicated by mapped QTL

Combined across panels, the QTL regions associated with adult copper resistance include a total of 1823 unique protein-coding genes. We identified potential candidate genes by searching among this list for genes with previous links to metal homeostasis, binding, metabolism, toxicity response, or transport, by executing QTL-focused, variant-by-variant association tests (Supplementary Figures S7–S9), and by examining variation in the estimated effects of founder haplotypes across QTL intervals (Supplementary Figures S7–S9). Of the 1823 genes, 10 genes have been previously linked to copper homeostasis, binding, chaperone activity, or development of specialized copper cells (Supplementary Table S4). Promising candidate genes include *Syx5*, *Grx1*, *CG11825*, *Ccs*, *Sod3*, and *CG5235*. *Syntaxin 5* (*Syx5*), encompassed by Q2, *Glutaredoxin 1* (*Grx1*), encompassed by Q9, and *CG11825*, encompassed by Q5, are all thought to play a role in copper ion homeostasis (Norgate *et al.* 2007, 2010; Mercer and Burke 2016). *Syx5* is required for normal uptake of cellular copper, and it plays a critical role in copper ion homeostasis in *D. melanogaster* that is independent of other copper transporter proteins such as *Ctr1A/B* (Norgate *et al.* 2010). Similarly, *Grx1* knockdown results in copper deficiency, and this gene may function as a mediator of copper transfer to chaperone proteins (Mercer and Burke 2016). *CG11825* has been identified as a candidate for copper ion homeostasis in *D. melanogaster* by Norgate *et al.* (2007), but functional testing is lacking for this gene under copper stress conditions. The gene copper chaperone for superoxide dismutase (*Ccs*), found under Q5, is an important chaperone protein that shuttles copper ions to *Sod1* under normal conditions (Culotta *et al.* 1997; Schmidt *et al.* 2000). *Ccs* was further supported as a promising candidate gene following variant-by-variant association tests of the QTL Q5 interval (Supplementary Figure S8). Genetic ablation of *Ccs* in *D. melanogaster* resulted in increased sensitivity to oxidative stress following paraquat exposure (Kirby *et al.* 2008); however, the effect of *Ccs* knockdown under copper stress conditions has not been assessed. Genes previously associated with copper ion binding include *Sod3* (Q6) and *CG5235* (Q8). While *Sod3* functions as an extracellular receptor for copper ions and is protective against oxidative stress (Blackney *et al.* 2014), the link between *CG5235* and copper is based only on prediction informed by GO (Gaudet *et al.* 2011).

In addition to these copper-associated genes, we identified 64 genes with functions related to homeostasis or detoxification of zinc, two genes involved with manganese regulation, and 19 genes involved in binding unspecified metals. Of particular interest among these genes are *Catsup* (Q3), *ZnT41F* (Q4), and *stl* (Q7), which are all linked to zinc transport or detoxification (Yepiskoposyan *et al.* 2006; Ozdowski *et al.* 2009; Lye *et al.* 2013; Navarro and Schneuwly 2017), *trpl* (Q5) and *DCP2* (Q8), which are hypothesized to be involved in manganese ion binding (Thurmond *et al.* 2019), and *swm* (Q3), *babo* (Q5), and *whd* (Q5), which are thought to be involved in binding of unspecified metal ions based on GO prediction (Gaudet *et al.* 2011; Thurmond *et al.* 2019). Similar to *Ccs*, the gene *trpl* was supported by variant-by-variant association tests of the Q5 interval (Supplementary Figure S8), further suggesting this gene is a promising candidate for functional testing under copper conditions.

The three QTL associated with copper treatment-specific developmental viability spanned a total of 247 unique protein-coding genes. Of these genes, none had functions previously linked to copper. However, eight genes were associated with zinc ion binding, and two were linked to metal ion binding through GO prediction (Supplementary Table S4; Gaudet *et al.* 2011; Thurmond *et al.* 2019). Most notable among the genes identified by treatment-specific developmental viability QTL was *mekk1* (Q15), which was demonstrated though gene knockdown to be the primary activator of JNK signaling under cadmium stress in *Drosophila* S2 cells (Ryabinina *et al.* 2006). Although the Q15 developmental viability QTL overlaps with the adult copper resistance QTL Q11, *mekk1* is only present within the interval implicated by Q15. Given that *mekk1* is within 11.2 kb of the Q11 2-LOD drop interval, this gene may still be a plausible candidate for adult copper resistance.

### Differential gene expression due to treatment and resistance class

Allelic effects on variation in complex traits are commonly mediated by regulatory variation (Roelofs *et al.* 2006; Ruden *et al.* 2009; Boyle *et al.* 2017; GTEx Consortium *et al.* 2018). We used an RNA sequencing approach to examine the effects of copper stress on gene regulation and to assess any differences in this response between genotypes with high or low adult copper resistance. We sequenced mRNA from whole females from six high (79–98%) and four low (0–18%) adult copper resistance strains from the B panel following a 9-h exposure to control (water) and 50 mM CuSO_4_ conditions. A primary goal was to determine whether there are consistent differences in gene expression between high and low resistance classes of strains when exposed to copper stress, so we treated each strain as a replicate of the high and low resistance classes. We performed differential expression analysis by first testing whether an interaction between treatment and resistance class influenced gene expression (interaction model: ∼TRT × RES *vs* reduced model: ∼TRT + RES). We followed this initial analysis with three subsequent analyses to test the additive effects of treatment and resistance class on gene expression: (1) Full model: ∼TRT + RES *vs* reduced model: ∼1), (2) Treatment Model: ∼TRT + RES *vs* reduced model: ∼RES), and (3) Resistance Class Model: ∼TRT + RES *vs* reduced model: ∼TRT. The treatment and resistance class models allowed us to identify specific genes that were influenced primarily by either of these two main effects.

**Figure 6:**
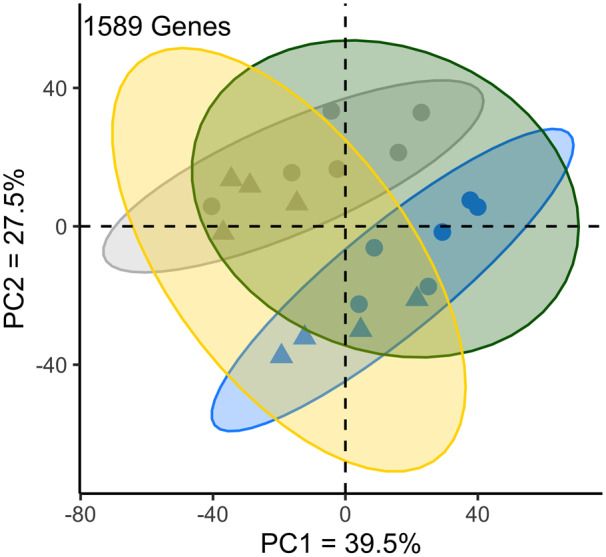
Differentially expressed genes cluster by treatment and resistance class. Principal components analysis of significantly differentially expressed genes (quantile-normalized filtered TPM) identified by the full model (full model: ∼TRT + RES *vs* reduced model: ∼1). The effect of treatment was pronounced among samples, while the effect of resistance level was more subtle. Ellipses indicate the equivalent of a 95% confidence interval. Blue indicates copper-exposed samples, gray indicates control-exposed samples, green indicates resistant strains, yellow indicates sensitive strains. Triangle points indicate sensitive strains; circles indicate resistant strains.

The interaction between treatment (control *vs* 50 mM CuSO_4_) and resistance class was not significant at a 5% FDR or at a relaxed FDR of 20%. Lack of a significant interaction is likely due to limited power as a result of small sample size. After additional filtering (see *Materials and Methods*), we identified 1589 genes that were DE across treatment and adult copper resistance class with the full model (full model: ∼TRT + RES *vs* reduced model: ∼1). We used PCA with quantile-normalized filtered TPM data from these 1589 genes and found a pronounced effect of treatment on gene expression, with a more subtle effect of resistance class (Figure 6, Supplementary Table S5). The relatively subtle effect of resistance class on gene expression likely also contributes to the lack of a significant treatment by resistance class interaction.

To further explore how each of the main effects of the differential expression model influence gene expression, and to identify sets of genes that were influenced primarily by treatment or resistance class, we tested the effects of treatment and resistance class separately. Treatment alone (treatment model: ∼TRT + RES *vs* reduced model: ∼RES) primarily contributed to differential expression in 848 genes, and adult copper resistance alone (resistance model: ∼TRT + RES *vs* reduced model: ∼TRT) primarily contributed to differential expression in 466 genes. The vast majority of genes influenced by treatment and resistance class were included among the 1589 genes identified with the full model (92% of genes identified with the treatment model, 91% of genes identified with the resistance model). Of the 848 and 466 genes identified with the treatment and resistance class models, 58 genes were shared. The proportion of shared genes increased when a more relaxed significance threshold (20% FDR) was used in the treatment and resistance class models, and the estimated effects of resistance class and treatment on gene expression in each DE gene list were weakly positively correlated (Treatment Model: *R*^2^ = 8%; Resistance Class Model: *R*^2^ = 2%). While the DE gene lists attributed to treatment and resistance class are not fully independent, they represent sets of genes for which the primary source of variation is either treatment or resistance class.

To broadly characterize DE genes identified in the treatment and resistance class models, we performed GO analysis with FlyMine (Lyne *et al.* 2007) for each gene list separately. GO analysis of each complete DE gene list is summarized in Supplementary Table S6. Briefly, 111 GO terms were identified from the full model DE gene list and included terms related to cell organization, cell cycle, and metabolism among the top 10 (Supplementary Table S6). Enrichment for 23 GO terms including those related to cytoplasmic translation, ribosome biogenesis, and RNA processing was observed for the 848 genes influenced by treatment (Supplementary Table S6). Fifty-nine GO terms were identified from the 466 DE genes influenced by resistance class. Top among these GO terms were those related to ATP synthesis, cellular respiration, and mitochondrial function (Supplementary Table S6). GO analysis of this set of 58 genes revealed enrichment for female gamete generation [*GO:0007292*] (*P* = 0.006), and no genes had any apparent connection to copper or metal ion homeostasis. Notably, no enrichment was observed for any GO term related to metal ion homeostasis or detoxification when the total DE gene lists were considered (but see below).

Many of the DE genes influenced by treatment and/or resistance class fell within the QTL intervals for adult copper resistance or treatment-specific developmental viability (Figure 5). Of the 848 genes with DE due to treatment, 87 (11%) overlapped with QTL intervals, and of the 466 genes with DE due to resistance class, 62 genes (13.3%) overlapped with QTL intervals. Of the 58 genes shared between the treatment and resistance class models, 12 genes (20.6%) overlapped with QTL intervals (Figure 5). DE genes from the treatment and resistance class models were not more likely than expected by chance to fall within QTL intervals (χ^2^ = 14.5, df = 14, *P* = 0.41), although this does not preclude the possibility that those DE genes within mapped QTL are strong candidates that may contribute to copper resistance.

To further explore the influence of resistance class on gene expression, we calculated the average change in gene expression following copper exposure for each of the 1589 DE genes from the full model using the same filtered TPM data used for PCA above. The absolute values of these data were then log transformed to reduce spread, and the sign of the change in gene expression was restored by multiplying the result by 1 or −1.

**Figure 7:**
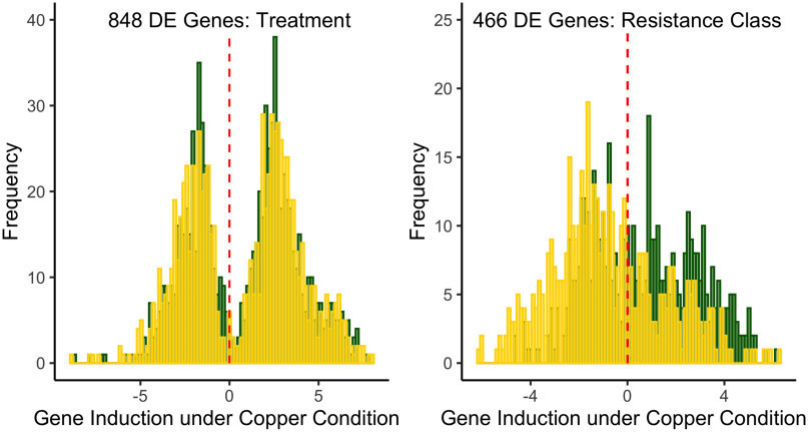
Copper-induced changes in gene expression vary by treatment and resistance class. Induction of genes under copper conditions with DE identified from the treatment model (A) and the resistance model (B). (A) The effect of treatment on DE highlights that roughly equal numbers of genes were induced or repressed under copper conditions (KS test: *D* = 0.05, *P* = 0.33). (B) Among DE genes identified by the resistance model, genes were more likely to be induced by copper exposure in sensitive strains compared to resistant strains (KS test: *D* = 0.27, *P* < 0.001). In each plot, yellow bars indicate gene expression in sensitive strains and green bars indicate gene expression in resistant strains.

Of the 848 DE genes identified in the treatment model, there was a roughly even split between copper-induced and copper-repressed genes, with no difference between the resistance classes [Kolmogorov–Smirnov (KS) test: *D* = 0.05, *P* = 0.33; Figure 7A]. Among the top 20 most highly induced genes under copper conditions in both resistant and sensitive classes were several MTs (*MtnA*, *MtnC*, *MtnD*, *MtnE*) as well as two genes that comprise a major iron storage complex (*Fer1HCH* and *Fer2LCH*). Because these genes and other genes with DE due to treatment were induced in sensitive and resistant strains to similar degree, we suggest that variation in sensitivity to copper is not due to a failure to induce expression of genes with protective functions against copper ions. Among the 466 DE genes identified in the resistance model, gene induction by copper was more frequently observed in sensitive strains compared to resistant strains (KS test: *D* = 0.27, *P* < 0.00001; Figure 7B). The top 20 most highly induced genes under copper conditions in sensitive strains included several genes that are involved in mitochondrial structure, function, and energy synthesis (*e.g.*, *Ald1*, *levy*, *sesB*, *Mpcp1*, *COX5A*, *ATPsynb*), suggesting more sensitive strains may be characterized by a greater susceptibility to oxidative stress.

### Cluster analysis of DE genes

The patterns of copper-influenced gene expression across treatment and resistance class observed in Figure 7 raise the question of whether there are co-regulated sets of genes that distinguish resistant and sensitive strains under copper and control conditions. For example, the presence of metal-associated genes among the those induced by treatment may signal a larger network of genes that are co-regulated in response to heavy metal stress. To identify any such co-regulated groups, we used Clust (Abu-Jamous and Kelly 2018) to identify non-overlapping clusters of genes from the 848 genes influenced by treatment and the 466 genes influenced by resistance class using quantile-normalized, filtered TPM data.

**Figure 8:**
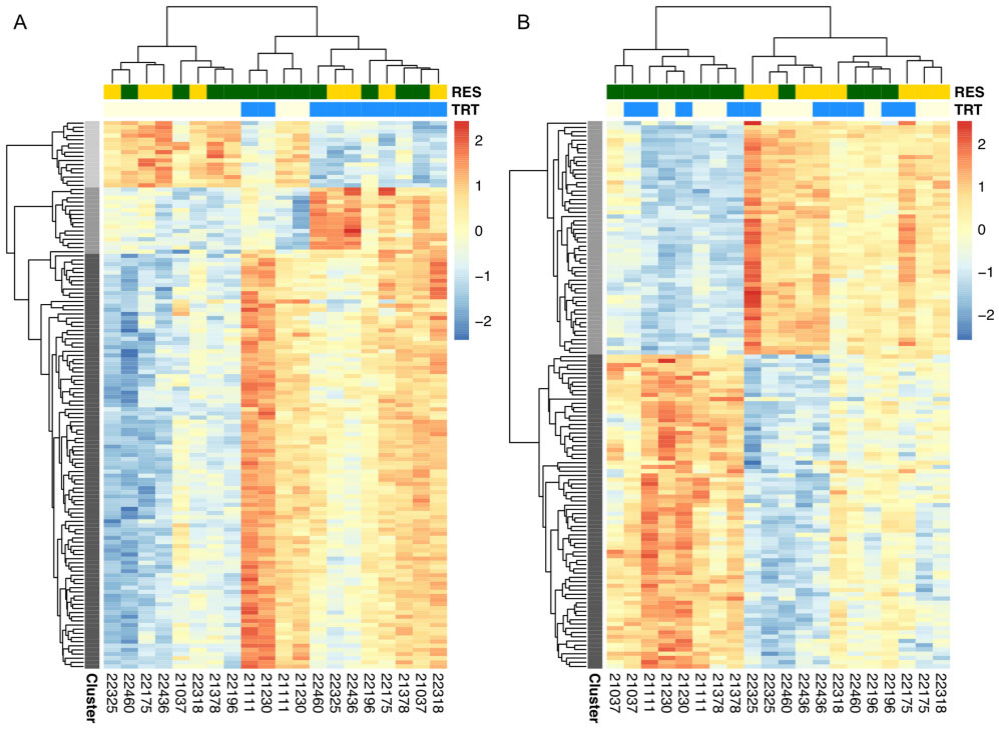
Heatmap of differentially expressed genes due to treatment and resistance class. Three clusters were identified among the genes with DE due to treatment (A), and two clusters were identified among genes with DE due to resistance class (B). Expression is presented following z-normalization of the 132 and 129 genes that clustered from the treatment gene set and the resistance class gene set, respectively. Cluster is mapped to the *y*-axis dendrogram [Cluster 1 (A, B) = dark gray, Cluster 2 (A, B) = medium gray, Cluster 3 (A) = light gray], and resistance class (green = resistant, yellow = sensitive) and treatment (blue = copper-exposed, cream = control) are mapped to the *x*-axis dendrogram.

Clust identified three clusters of co-regulated genes with DE due to treatment (Figure 8A, Supplementary Figure S11A). Treatment clusters 1 (101 genes) and 2 (17 genes) consisted primarily of genes that were induced by copper exposure, while treatment cluster 3 (17 genes) consisted primarily of genes that were copper-repressed (Figure 8A, Supplementary Figure S11A). The top GO terms for treatment clusters 1 and 3 revealed enrichment for genes involved in processes unrelated to metal ion homeostasis or response (*e.g.*, cell cycle, RNA processing; Supplementary Table S6). However, treatment cluster 2 was enriched for genes involved in iron import, transport, and detoxification of iron and inorganic compounds (Supplementary Table S6). Among these genes were *Fer1HCH* and *Fer2LCH*, which are primarily involved with iron storage, but may also interact with copper ions during protein assembly (Huard *et al.* 2013). Despite these GO terms suggesting treatment cluster 2 is enriched for genes that respond to toxic metal ion exposure, only one of the genes (*Gclc*) has been previously directly linked to copper (*Gclc* interacts with the copper transport proteins *Ctr1A* and *ATP7*; Mercer *et al.* 2016). Treatment clusters 1 and 3 included 17 and four genes, respectively, that were implicated by adult copper resistance-associated QTL. The gene *dnk* in treatment cluster 1 was also implicated by the treatment-specific developmental viability QTL Q15. Two of the genes (*CG11878* and *CG5506*) identified in treatment cluster 2 were implicated by adult copper resistance-associated QTL intervals. One gene, *CG5506*, was empirically demonstrated to interact with *Fer2HCH* (Guruharsha *et al.* 2011); however, neither gene has been previously associated with copper exposure.

**Figure 9:**
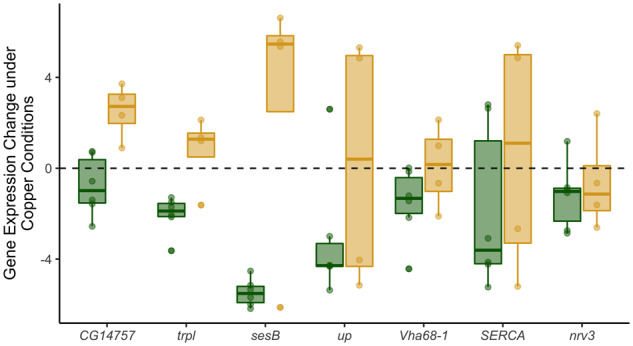
Copper-affected expression of genes involved in inorganic ion homeostasis that were included in resistance cluster 2. Resistant strains are shown in green, sensitive strains are shown in yellow.

Clust identified two clusters of co-regulated genes that were DE due to resistance class (Figure 8B, Supplementary Figure S11B). Resistance cluster 1 (75 genes) was primarily enriched for genes involved in cell cycle processes (Supplementary Table S6), and 11 genes were also implicated by adult copper resistance QTL. Resistance cluster 2 (56 genes) included genes that were more often copper-induced in sensitive strains and copper-repressed in resistant strains (Figure 8B, Supplementary Figure S11B). Resistance cluster 2 was enriched for two broader categories of GO terms including several related to muscle structure (*e.g.*, myofibril assembly [*GO:0030239*], *P* < 0.00001) and mitochondrial function and energy synthesis (*e.g.*, ATP metabolic process [*GO:0046034*], *P* < 0.00001). Resistance cluster 2 also included genes involved in inorganic ion homeostasis (*GO:0098771*), although enrichment for this GO term was weak (*P* = 0.05). Of the genes involved in inorganic ion homeostasis, three (*CG14757*, *trpl*, and *sesB*) are particularly noteworthy given that all three genes are copper-induced in sensitive strains and copper-repressed in resistant strains, and two have been previously linked to metal ion homeostasis (Figure 9). Exposure of the *Drosophila* S2 cell line to 2 mM CuSO_4_ resulted in increased expression of *CG14757*, indicating that this gene is responsive to copper stress (Norgate *et al.* 2007); however, its exact function relative to the toxic effects of copper has not been elucidated. The gene *trpl* is predicted to be involved in manganese ion binding (Thurmond *et al.* 2019) and was included in the adult copper resistance-associated QTL Q5 we mapped. *sesB* is a mitochondrial transporter gene that was demonstrated to be important for protection against oxidative stress through gene knockdown in *D. melanogaster* (Terhzaz *et al.* 2010). Other genes included in this group (*up*, *SERCA*, and *nrv3*; Figure 9) are involved in transport of calcium, sodium, and potassium (Domingo *et al.* 1998; Gaudet *et al.* 2011) or are thought to be involved in ATP metabolism (*Vha68-1*) (Thurmond *et al.* 2019). In addition to *trpl*, two other genes from resistance cluster 2 were implicated by adult copper resistance QTL.

### RNAi knockdown of candidate genes associated with adult copper survival

Several genes with links to copper or metal ion homeostasis were implicated by QTL or were DE due to treatment or resistance class (see above) (Supplementary Table S3). We chose 16 genes to functionally test using RNAi knockdown. QTL-implicated genes included *Catsup* and *swm* (Q3), *ZnT41F* (Q4), *CG11825*, *whd*, *babo*, and *Ccs* (Q5), *stl* (Q7), *DCP2* and *CG5235* (Q8). We also tested *trpl* (Q5), which, along with *Mvl*, was among the DE genes influenced by resistance class. Because *Ccs* and both *Sod1* and *Sod2* closely interact, we tested *Sod1/2* even though these genes were not implicated by either QTL or RNAseq. From genes with DE due to treatment, we tested *MtnC* and *CG10505*. Of this set of genes, only *Sod1*, *MtnC*, and *Mvl* have been previously specifically linked to copper stress (Calap-Quintana *et al.* 2017). *Ccs*, *CG5235*, *Sod1*, *CG11825*, and *CG10505* are all associated with copper transport or binding. The remaining candidate genes (*trpl*, *DCP2*, *whd*, *stl*, *swm*, *babo*, *Catsup*, and *ZnT41F*) have not been experimentally linked to copper stress but are associated with metal ion binding or homeostasis.

Genes were tested using TRiP UAS RNAi strains (Perkins *et al.* 2015) that were crossed to a background with a ubiquitously expressed Gal4-expressing driver resulting in knockdown in the whole animal throughout all developmental stages, and to a background with an adult, anterior midgut-specific Gal4-expressing driver (Buchon *et al.* 2013). Copper absorption occurs in the copper-accumulating region of the middle midgut (Filshie *et al.* 1971; Calap-Quintana *et al.* 2017); however, the majority of candidate genes we tested (*trpl*, *Ccs*, *CG10505*, *Mvl*, *swm*, *babo*, *Catsup*, *CG11825*, *Sod1/2*, and *ZnT41F*) are expressed throughout the midgut, including the anterior midgut region (Buchon *et al.* 2013).

**Figure 10:**
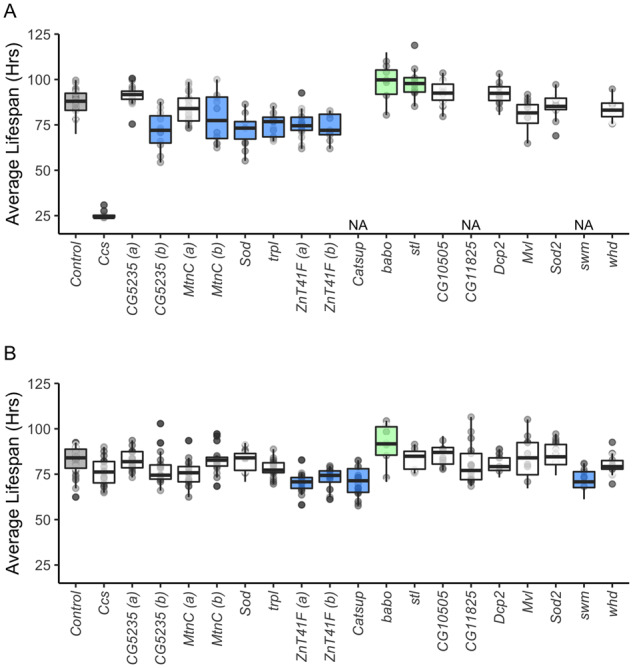
RNAi knockdown of candidate genes. Average lifespan of TRiP UAS RNAi knockdown strains crossed to a ubiquitous Gal4-expressing driver (A) and to an anterior-specific Gal4-expressing driver (B). (A) Increased susceptibility was observed with knockdown of *Ccs*, *CG5235* (b), *MtnC* (b), *Sod*, *trpl*, and *ZnT41F* with the ubiquitous Gal4 driver. Knockdown of *babo* resulted in increased resistance to copper toxicity relative to the control. (B) Knockdown in the anterior midgut of *Catsup*, *swm*, and *ZnT41F* resulted in increased susceptibility to copper toxicity, while knock down of *babo* increased resistance relative to the control. In each plot, gray shading indicates the control, green shading indicates increased resistance to copper, blue shading indicates decreased resistance, and no shading indicates lack of a significant difference based on an experiment-wide *a* = 0.05. Three candidate gene TRiP strains (*swm*, *Catsup*, and *CG11825*) produced too few flies to test when crossed to the ubiquitous Gal4-expressing driver and were thus excluded from our analysis. We tested multiple TRiP UAS RNAi strains for genes *CG5235* [*CG5235* (a), *CG5235* (b)], *MtnC* [*MtnC* (a), *MtnC* (b)], and *ZnT41F* [*ZnT41F* (a), *ZnT41F* (b)] to assess the consistency in the effect of gene knockdown on copper survival (Supplementary Table S3).

In general, more genes influenced copper resistance when they were knocked down in the whole animal compared to solely in the anterior midgut (Figure 10). Of the candidate genes with known associations with copper, *Ccs*, *CG5235* (b), *MtnC* (b), and *Sod1* reduced copper resistance relative to the control when knocked down in the whole animal using the ubiquitous driver (Figure 10A). Inconsistent effects of ubiquitous *CG5235* knockdown may be influenced by vector efficiency; the knockdown vector for *CG5235* (a) is a long dsRNA vector (VALIUM10), while the knockdown vector for *CG5235* (b) is a shRNA vector (VALIUM20). Both TRiP strains for *MtnC* used the same vector (VALIUM20) (Perkins *et al.* 2015); however, these two strains target *MtnC* at different locations within the gene, and knockdown efficiency may differ between the two sites. Off-target effects and leaky gene expression are additional potential sources of error in RNAi knockdown experiments. However, TRiP RNAi strain attributes have been shown to improve efficiency with limited off-target effects (Ni *et al.* 2009, 2011; Perkins *et al.* 2015). The majority of TRiP strains used in our study implement short hairpin RNAs with the VALIUM20 vector, which has been demonstrated to have strong knockdown effects (Ni *et al.* 2011). Knockdown of copper-associated genes in the anterior midgut did not influence copper resistance relative to the control, suggesting that reduced expression of *Ccs*, *CG5235*, *Sod1*, *CG11825*, and *CG10505* in this limited region of the midgut does not hinder the fly’s ability to cope with copper stress (Figure 10B).

The candidate gene *ZnT41F* consistently reduced copper resistance relative to control when knocked down in the whole animal and in the anterior midgut. While *ZnT41F* was previously shown to indirectly affect zinc homeostasis (Yin *et al.* 2017), the role it plays in copper ion homeostasis has not been described. Similarly, *Catsup* and *swm*, which have not been previously linked to copper, reduced copper resistance when knocked down in the anterior midgut. That knockdown of these genes in the whole animal did not influence copper resistance suggests these genes interact with copper soon after ingestion, although this would require additional follow-up to confirm. Interestingly, knockdown of *babo* in both the whole animal and the anterior midgut increased copper resistance relative to the controls (Figure 10). Knockdown of *stl* in the whole animal had a similar effect. Both genes are predicted to be involved in metal ion binding (Gaudet *et al.* 2011; Thurmond *et al.* 2019), but any additional evidence linking them to the detoxification of heavy metal ions under stressful conditions is lacking.

## Discussion

### Variation in heavy metal stress is influenced by a complex genetic architecture

*Drosophila melanogaster* has been an important model for elucidating the roles of genes involved in the response to copper and other heavy metals (*e.g.*, Egli *et al.* 2003, 2006a, 2006b; Balamurugan *et al.* 2004, 2007; Yepiskoposyan *et al.* 2006; Calap-Quintana *et al.* 2017). In our study, we used this model to investigate the role of allelic and expression variation in resistance to the heavy metal copper. We used a combination of QTL mapping and RNA sequencing to characterize allelic and gene expression variation that influences resistance to copper stress in strains from the multiparental DSPR mapping panel. In comparison with previous reports investigating the genetic architecture of copper and resistance to other heavy metals in plants (Macnair 1993; Selby and Willis 2018), the genetic architecture of copper resistance in *D. melanogaster* appears to be more complex. Where one to three QTL were identified for heavy metal resistance in several plant species including *Mimulus guttatus*, wheat, and corn (Allen 1971; Macnair 1983, 1993; MacNair *et al.* 1993; Bálint *et al.* 2007; Selby and Willis 2018), we identified 12 QTL that underlie variation in adult copper resistance (Figure 5A), and found that the diverse DSPR strains varied widely in survival following exposure to copper stress (Figure 1).

Part of the difference in apparent complexity underlying the response to copper is likely due to the higher power of our mapping panel, which employs a much larger number of genetically diverse strains coupled with higher genetic marker density compared to the mapping populations used in many plant studies (*e.g.*, Courbot *et al.* 2007; Willems *et al.* 2007; King *et al.* 2012a). Secondly, the structure of the DSPR may be particularly conducive for detecting allelic variation in genes that influence the response to copper stress given the global sampling of founder strains used to generate the DSPR (King *et al.* 2012a), which may capture more of the natural variation for copper resistance than that present in any one natural population. Third, in contrast to natural populations which are often of interest because of their proximity to heavy metal pollution (*e.g.*, Allen 1971; Macnair 1983; Ramirez *et al.* 2005; Turner *et al.* 2010; Wuana and Okieimen 2011; Wright *et al.* 2015; Arnold *et al.* 2016), the DSPR is likely naïve to any form of heavy metal selection or stress. Strong selection for heavy metal resistance could reduce variation at causative genes and lead to an apparent reduction in the complexity of resistance (Arnold *et al.* 2016).

The level of genetic complexity for copper resistance described in our study is consistent with reports of metal resistance in flies, yeast, and worms where measures of resistance were conducted in other heavy metal-naïve mapping populations. The DGRP (Mackay *et al.* 2012), another large *D. melanogaster* mapping panel, was used to demonstrate a complex genetic architecture for heavy metal exposure through GWA (genome-wide association) and extreme QTL mapping (*i.e.*, sequencing and comparing pools of individuals with divergent phenotypes) in adult and developing life stages (Montgomery *et al.* 2014; Zhou *et al.* 2016, 2017). In these studies, tens to hundreds of genes have been implicated in natural genetic screens for lead (Zhou *et al.* 2016, 2017), cadmium (Zhou *et al.* 2017), and methylmercury (Montgomery *et al.* 2014). In *Saccharomyces cerevisiae* using several extreme QTL mapping pools, Ehrenreich *et al.* (2010) demonstrated that more than 20 distinct loci were associated with resistance to cadmium and nearly 40 loci were associated with copper resistance. In *Caenorhabditis elegans*, Evans *et al.* (2018) found 4, 6, and 6 QTL associated with the response to cadmium, copper, and silver, respectively.

In addition to a complex genetic architecture underlying the response to any one metal stressor, it is also possible that genes that are linked to one metal may play a role in the response to other metals through pleiotropic effects or as members of gene networks. Previous studies in yeast and worms have found limited evidence of pleiotropy underlying the response to multiple chemical stressors (Ehrenreich *et al.* 2010; Evans *et al.* 2018). However, we noted that several promising candidate genes associated with copper in our study had been previously linked to or were predicted to interact with metals other than copper [*e.g.*, *swm* (Q3), *babo* (Q5), and *stl* (Q5); Figure 10, Supplementary Table S3]. Although we did not measure resistance to multiple heavy metal stressors in our study, RNAi knockdown of genes linked to other metals did impact copper resistance (Figure 10). Pleiotropic gene effects inferred from our RNAi analyses may be the result of metal-sensitive genes responding to a generalized set of cytotoxic effects stemming from production of ROS caused by heavy metal toxicity (Uriu-Adams and Keen 2005). This hypothesis is further supported by evidence of copper-induced expression of genes involved in oxidative stress response in sensitive strains that are repressed in resistant strains (*e.g.*, *sesB*, Figure 9). However, additional tests of the response of DSPR strains to a diverse set of heavy metals is needed to fully understand whether the non-copper candidate genes we identified have correlated effects on resistance to other heavy metals.

Additional functional testing of the candidate genes highlighted in our study would also help illuminate the role of natural variation in the candidate genes we identified in copper resistance in wild populations. Our study presents evidence that RNAi knockdown of a set of candidates alters copper resistance; however, this approach does not allow us to investigate the effects of naturally occurring allelic variation, and only indirectly points to the likelihood these genes harbor segregating variation impacting response to copper. Reciprocal hemizygosity experiments (Stern 2014) would allow us to test putatively functionally distinct alleles present among the DSPR founder strains (Supplementary Figure S6), and genome editing tools such as CRISPR-Cas9 can be used to generate null mutations of candidate genes in high and low resistance DSPR strains.

### Consistency in the genetic architecture of copper resistance across life stages

Genes that are involved in copper homeostasis in *D. melanogaster* adults have in some cases also been shown to regulate copper in larvae. For example, exposure of larvae to CuSO_4_ induces expression of MTs (Egli *et al.* 2003, 2006b), and we demonstrated that copper induces higher MT expression in adults (Supplementary Table S5). Knockdown of copper transporter genes in the CTR family alters copper homeostasis in both larvae and adult flies as well (Zhou *et al.* 2003; Turski and Thiele 2007). Our goal was to understand the relationship between adult copper resistance and the effect of copper stress on development in a set of genetically diverse *D. melanogaster* strains.

Similar to previous reports, we found that copper stress delayed development and reduced viability (Zhou *et al.* 2003; Bahadorani and Hilliker 2009; Pölkki 2016) although to differing degrees among the DSPR strains (Figure 3), suggesting that as with adult copper resistance, treatment-specific development time and developmental viability are genetically variable. Despite the lack of a statistically significant correlation between the developmental responses to copper stress and adult copper resistance (Figure 4), we did observe evidence of partially shared genetic architectures between treatment-specific developmental viability and adult copper resistance (Figure 5B). Additional testing would be needed to determine whether the same genes implicated by developmental viability QTL Q15 and adult copper resistance QTL Q11 influence copper resistance at each life stage.

Because the ecology of the developing and adult stages of *D. melanogaster* are quite distinct, that copper resistance might be influenced by largely life stage-specific mechanisms is not unexpected. For example, *D. melanogaster* adults and larvae avoid copper-supplemented food when given the opportunity (Balamurugan *et al.* 2007; Bahadorani and Hilliker 2009); however, in natural populations, higher mobility of adults would allow the adult life stage to avoid heavy metal contaminated food more effectively. Life-stage specific genetic architectures were observed in *D. melanogaster* for cold tolerance (Freda *et al.* 2019), and the decoupling of the genetic mechanisms that influence survival and fitness have been reported in diverse organisms with complex life cycles (Moran 1994; Ragland and Kingsolver 2008). However, a number of other factors including the large difference in copper dose and the nature of the response tested at each life stage may obscure or complicate the relationship between the developmental and adult responses we observed. We used a much lower dose in our assessment of the effect of copper on development time and developmental viability compared to the adult copper resistance phenotype, and differences in dose can alter the genetic architecture for a trait. For example, in the yeast *S. cerevisiae*, Wang and Kruglyak (2014) demonstrated that the overall genetic architecture of haloperidol resistance was dose-dependent. While one QTL was consistently detected for each of the five doses tested, several QTL were only detected at a single dose (Wang and Kruglyak 2014). With the added complexity of assessing the effects of copper in different life stages, it is difficult to fully determine whether the effects of copper in the adult and developmental assays are analogous. Further confounding this comparison, our adult copper assay was implemented over a 48-h time period in contrast to exposing developing flies from egg to adult to copper over a period of 30 days at most. The harmful effects of copper on development may be constant or variable across different stages (egg, larvae, pupation), and this represents an area of ongoing research.

### Copper sensitivity is influenced by gene expression variation and behavior

Differences in expression levels of genes that have protective functions against toxins, or that are co-opted by toxins can lead to variation in resistance levels. For instance in humans, natural variation in expression levels of the gene *CMG2* is associated with variation in resistance to anthrax (Martchenko *et al.* 2012), and in the fungus *Suillus luteus*, selection pressure from heavy metal pollution quickly led to copy number variation in transport genes with protective functions against heavy metal toxicity (Bazzicalupo *et al.* 2019). Sensitivity to copper in adult *D. melanogaster* DSPR strains does not appear to be due to insufficient expression of genes involved with copper or metal detoxification such as MTs or CTR family transporters (Supplementary Table S5). Instead, we found that genes associated with metabolism and mitochondrial function were copper-induced in sensitive strains and copper-repressed in resistant strains (Figure 9). Stress caused by toxic levels of copper ions results in overproduction of ROS, which can alter energy production and metabolism (Uriu-Adams and Keen 2005; Tchounwou *et al.* 2008; Quijano *et al.* 2016). Negative effects of the misregulation of or overexposure to copper and other heavy metals such as zinc, lead, mercury, and arsenic through disease (Giacoppo *et al.* 2014; Liddell and White 2018; Umair and Alfadhel 2019) or because of pollution have been associated with impaired or altered mitochondrial function (Belyaeva *et al.* 2011, 2012; Jia *et al.* 2015). It is therefore possible that variation in metabolic function among the DSPR strains is one of the underlying contributors to variation in copper resistance. Given that we also observed that sensitive strains are slightly more likely to consume copper in larger amounts in a 24-h period compared to resistant strains (Figure 2), sensitive strains may be under greater metabolic stress as they cope with exposure to behaviorally mediated higher levels of ingested copper. Copper resistance in *D. melanogaster* may not be simply a function of how well the organism is able to detoxify food; more likely, copper resistance is a combination of behavioral aversion to copper and the metabolic stress induced by the amount of metal consumed in addition to detoxification ability.

In general, food consumption rate has a complex genetic basis in *D. melanogaster* (Garlapow *et al.* 2015), and when given a choice, both *D. melanogaster* adults and larvae tend to avoid copper-supplemented food at much lower concentrations relative to those tested in this study (Balamurugan *et al.* 2007; Bahadorani and Hilliker 2009). Bahadorani and Hilliker (2009) showed that adult copper avoidance was observed at 1 mM CuSO_4_, and avoidance in third-instar larvae was observed at 0.25 mM CuSO_4_ (Balamurugan *et al.* 2007). Similarly, adult *D. melanogaster* avoid pupation and oviposition on copper-supplemented food (Bahadorani and Hilliker 2009). While this behavioral component likely plays an important role in mediating copper stress in natural populations, these studies focused on only one or few genetic strains, making it difficult to extrapolate how a genetically variable population would behave in response to copper. The correlation between adult copper resistance and copper food consumption in the 100 DSPR strains tested in our study suggests that variation in copper avoidance may play an important role in overall adult copper resistance. At this point, the specific relationship between copper consumption rates, metabolic stress, and genetic resistance to copper has not been characterized, but doing so in future studies has potential to more clearly define resistance to ingested toxins compared to an assessment based solely on survival. Important remaining questions include whether behavioral avoidance and sensitivity to heavy metals are influenced by variation in chemosensory detection ability (*e.g.*, Arya *et al.* 2015; He *et al.* 2016) or variation in preference for metal-supplemented food (*e.g.*, Highfill *et al.* 2019). Addressing these questions with a large panel such as the DSPR will help support our efforts to characterize the relationship and potential interaction between behavior and genetic capacity for copper resistance.

### Conclusions

Copper resistance in *D. melanogaster* is genetically complex, is influenced by allelic and expression variation as well as by variation in behavioral avoidance of copper, and may be controlled by distinct sets of loci in different life stages. Several genes that have known copper-specific functions as well as genes that are involved in the regulation of other heavy metals were identified as potential candidates for variation in adult copper resistance and treatment-specific developmental viability. We demonstrated that nine of these candidates influenced adult copper resistance, providing evidence of pleiotropic effects of genes previously thought to be associated with other heavy metals. Copper is just one of many heavy metals that pollute the environment with negative impacts on humans, fungi, plants, and insects at a global scale. Understanding the complexity of the genetic basis of copper resistance and the potential sources of variation that interact with resistance is important for understanding the diverse mechanisms through which copper pollution can negatively impact organisms.
